# Response time variability under slow and fast‐incentive conditions in children with ASD, ADHD and ASD+ADHD

**DOI:** 10.1111/jcpp.12608

**Published:** 2016-07-28

**Authors:** Charlotte Tye, Katherine A. Johnson, Simon P. Kelly, Philip Asherson, Jonna Kuntsi, Karen L. Ashwood, Bahare Azadi, Patrick Bolton, Gráinne McLoughlin

**Affiliations:** ^1^ King's College LondonMRC Social Genetic and Developmental Psychiatry CentreInstitute of Psychiatry, Psychology & NeuroscienceLondonUK; ^2^ King's College LondonChild & Adolescent PsychiatryInstitute of Psychiatry, Psychology & NeuroscienceLondonUK; ^3^ Melbourne School of Psychological SciencesUniversity of MelbourneMelbourneVic.Australia; ^4^ School of Electrical and Electronic EngineeringUniversity College DublinDublinIreland

**Keywords:** Autism spectrum disorder, attention deficit hyperactivity disorder, cognition, comorbidity, reaction time variability

## Abstract

**Background:**

Attention deficit hyperactivity disorder (ADHD) and autism spectrum disorder (ASD) show significant behavioural and genetic overlap. Both ADHD and ASD are characterised by poor performance on a range of cognitive tasks. In particular, increased response time variability (RTV) is a promising indicator of risk for both ADHD and ASD. However, it is not clear whether different indices of RTV and changes to RTV according to task conditions are able to discriminate between the two disorders.

**Methods:**

Children with ASD (*n* = 19), ADHD (*n* = 18), ASD + ADHD (*n* = 29) and typically developing controls (TDC;* n* = 26) performed a four‐choice RT task with slow‐baseline and fast‐incentive conditions. Performance was characterised by mean RT (MRT), standard deviation of RT (SD‐RT), coefficient of variation (CV) and ex‐Gaussian distribution measures of Mu, Sigma and Tau.

**Results:**

In the slow‐baseline condition, categorical diagnoses and trait measures converged to indicate that children with ADHD‐only and ASD + ADHD demonstrated increased MRT, SD‐RT, CV and Tau compared to TDC and ASD‐only. Importantly, greater improvement in MRT, SD‐RT and Tau was demonstrated in ADHD and ASD + ADHD from slow‐baseline to fast‐incentive conditions compared to TDC and ASD‐only.

**Conclusions:**

Slower and more variable RTs are markers of ADHD compared to ASD and typically developing controls during slow and less rewarding conditions. Energetic factors and rewards improve task performance to a greater extent in children with ADHD compared to children with ASD. These findings suggest that RTV can be distinguished in ASD, ADHD and ASD + ADHD based on the indices of variability used and the conditions in which they are elicited. Further work identifying neural processes underlying increased RTV is warranted, in order to elucidate disorder‐specific and disorder‐convergent aetiological pathways.

## Introduction

Attention deficit hyperactivity disorder (ADHD) and autism spectrum disorder (ASD) are common and highly heritable childhood‐onset disorders. Although ASD and ADHD are separate diagnoses, they share some behavioural and genetic features (Rommelse, Geurts, Franke, Buitelaar, & Hartman, 2011; Ronald, Happé, & Plomin, 2008). This raises the question as to whether ASD and ADHD share some common aetiology. The genetic and neurobiological underpinnings of these disorders are complex and new strategies have been proposed to identify cognitive and brain markers that may increase understanding of the pathophysiological underpinnings of these disorders (Rommelse et al., 2011; Tye, McLoughlin, Kuntsi, & Asherson, 2011). In this paper, we sought to investigate whether measures of response time variability (RTV) differentiate cases of ASD, ADHD and ADHD + ASD.

Children with ASD and with ADHD perform in a similar way on a range of cognitive tasks, yet deficits specific to a ‘core’ cognitive function have been difficult to demonstrate, likely to reflect high phenotypic and aetiological heterogeneity. A promising finding, however, is that increased intraindividual variability (IIV), as indexed by RTV, has been shown in both ASD and ADHD (for review, see Karalunas, Geurts, Konrad, Bender, & Nigg, 2014; Kuntsi, 2014) and may capture a proportion of the genetic influences that are shared between ADHD and ASD symptoms (Pinto, Rijsdijk, Ronald, Asherson, & Kuntsi, 2016). The lack of specificity could indicate that IIV is a common correlate of many psychiatric disorders (e.g. Kaiser et al., 2008) or related to shared genetic risk across disorders (Gottesman & Gould, 2003). Additionally, increased IIV may relate to or index liability for symptom dimensions that are shared across existing diagnostic boundaries, indicating a ‘transdiagnostic phenotype’ (Karalunas et al., 2014). A recent meta‐analysis published in JCPP suggested that children with ASD‐only show increased RTV when children with co‐occurring ADHD symptoms are included in the analysis (Karalunas et al., 2014), supported by our own work (Tye, Asherson, et al., 2014), which suggests that IIV indexes liability for high ADHD symptoms across diagnostic boundaries. One method for furthering our understanding of the nature of RTV in these disorders is to investigate variables that exacerbate or attenuate RTV. There is limited work, however, exploring the reliability of this finding across different task conditions and indices of RTV.

Current theories of ADHD suggest that suboptimal arousal may underlie increased RTV (Castellanos et al., 2005; Sergeant, 2000). The state regulation hypothesis is supported by the finding that task performance improves under conditions that optimise arousal in individuals with ADHD, such as an increased event rate (Kuntsi, Andreou, Ma, Borger, & van der Meere, 2005), rewards (Slusarek, Velling, Bunk, & Eggers, 2001) or both (Andreou et al., 2007; Kuntsi, Wood, Van der Meere, & Asherson, 2009), although findings are inconsistent and differ by task type and the performance measure used (Epstein et al., 2011). In ASD, there is some evidence of a state regulation deficit resulting in overarousal (although see Geurts et al., 2008), supported by increased RTV with faster event rates in adults with ASD (Raymaekers, van der Meere, & Roeyers, 2004). A direct comparison of the effect of variable event rates in ASD and ADHD revealed no support for a state regulation deficit in either disorder, despite individuals with ADHD demonstrating increased RTV overall (Raymaekers, Antrop, Van der Meere, Wiersema, & Roeyers, 2007). Altered reward processing has also been implicated in both disorders (Demurie, Roeyers, Baeyens, & Sonuga‐Barke, 2011), although relative improvements in performance with reward in ASD may be due to the presence of co‐occurring ADHD symptoms (Luman, Van Meel, Oosterlaan, Sergeant, & Geurts, 2009).

There are several properties of RTV that are likely to relate to different aspects of information processing that may allow for more defined characterisation of RTV manifestations in ASD and ADHD. In ADHD, RTV appears to reflect RTs in the exponential tail of the distribution as captured by the ex‐Gaussian parameter ‘Tau’ (Epstein et al., 2011; Kofler et al., 2013; Tamm et al., 2012), attributed to an increased frequency and higher magnitude of extremely long RTs (Leth‐Steensen, King Elbaz, & Douglas, 2000). There is evidence for an effect of event rate on tau, yet there is only partial support a greater improvement under faster conditions in ADHD compared to controls (Epstein et al., 2011). It is therefore important to separate Gaussian and ex‐Gaussian variability, and variability due to occasional slow responses, in order to capture these alternate processes in ASD and ADHD under different conditions.

In this study, task performance was measured in comprehensively assessed children with ASD, ADHD, ASD + ADHD and typically developing controls, using the ‘Fast task’ (Kuntsi et al., 2005), which involves a faster and overtly rewarding task that can be compared to a slower baseline condition. This task enables a test of whether ASD, ADHD and ASD + ADHD are characterised by increased RTV, whether this is specific to certain properties of RTV and whether any identified specificity is stable across conditions. Here we test the hypotheses that (a) slower and more variable RTs are demonstrated in children with ADHD, compared to ASD and typically developing controls in the slow‐baseline condition; (b) a faster event rate and incentives lead to greater improvement in speed and RTV in children with ADHD, compared to ASD and typically developing controls; and, more generally, (c) that children with ASD + ADHD present as an additive co‐occurrence of the unique disorders, as indicated by increased RTV in children with high ADHD symptoms regardless of categorical diagnosis.

## Methods

### Participants

Nineteen male participants with ASD, 18 with ADHD, 29 with ASD and ADHD, and 26 typically developing controls (TDC) took part in the study. The age range was 8–13 years; there was no significant difference in age across groups (Table 1). All participants were required to have an IQ > 70, normal or corrected‐to‐normal vision, and not to be taking any medication except for stimulants, which had to be interrupted 48 h prior to testing sessions. Exclusion criteria included nonfluent English, specific medical disorders, other comorbid psychiatric disorder (not including ODD), history of traumatic brain injury and a diagnosis of epilepsy.

**Table 1:** Clinical and demographic characteristics

	Diagnosis								*F*	*p*	Post hoc
	TDC (*n *=* *26)		ASD (*n *=* *19)		ADHD (*n *=* *18)		ASD + ADHD (*n *=* *29)				
	Mean	*SD*	Mean	*SD*	Mean	*SD*	Mean	*SD*			
Age	10.56	1.79	11.69	1.70	10.48	1.91	10.53	1.69	2.20	.093	n.s.d.
Verbal IQ	120.00	14.40	113.79	23.87	105.94	18.47	110.41	15.67	2.48	.066	n.s.d.
Performance IQ	115.73	13.89	111.05	13.31	101.67	11.60	106.72	11.97	4.86	.004	TD > ADHD
Full‐scale IQ	120.04	13.42	115.68	15.73	104.11	14.23	109.72	13.41	5.31	.002	TD > ASD + ADHD, ADHD
SCQ	3.88	3.54	20.11	6.42	10.89	5.36	24.79	5.71	81.12	<.001	TD < ADHD < ASD < ASD + ADHD
Conners DSM‐Inattentive	56.08	11.05	67.11	14.13	83.94	7.41	80.21	11.59	29.85	<.001	TD < ASD < ASD + ADHD, ADHD
Conners DSM‐Hyperactive	58.88	17.02	66.11	12.99	87.89	3.25	84.00	7.63	32.76	<.001	TD, ASD < ASD + ADHD, ADHD

n.s.d = nonsignificant difference.

The participants were recruited from outpatient neurodevelopmental clinics and local parent support groups in southeast London. All participants had a clinical diagnosis made according to ICD‐10 criteria (autism, Asperger's syndrome, ADHD combined type) and then underwent systematic and rigorous clinical assessment to confirm pure or comorbid research diagnosis (see Tye, Asherson, et al., 2014). All cases were initially evaluated with Conners’ 3rd Edition Parent Rating Scale short form (Conners, 2008) and Social Communication Questionnaire (SCQ; Rutter, Bailey, & Lord, 2003). Cases of ASD were diagnosed using the Autism Diagnostic Interview–Revised (ADI‐R; modified criteria IMGSAC, 1998) and the Autism Diagnostic Observation Schedule (ADOS‐G; Gotham, Risi, Pickles, & Lord, 2007). Cases of ADHD were diagnosed using Parent Account of Childhood Symptoms (PACS; Taylor, Schachar, Thorley, & Wieselberg, 1986), which has been extensively used by the IMAGE consortium (Chen et al., 2008). Comorbid ASD + ADHD cases met full diagnostic criteria for ASD using the ADI‐R/ADOS and full diagnostic criteria for ADHD using the PACS.

The TDC group consisted of children recruited through local schools and forums. TDC were assessed with the Strengths and Difficulties Questionnaire (SDQ; Goodman, 1997), SCQ and Conners’ questionnaires and were not included if they had any psychiatric diagnosis (see Tye, Asherson, et al., 2014). A medical ethics committee approved the study protocol. Written parental consent was given before the experiment began.

### Task

The ‘Fast task’ has been used in several previous studies of ADHD (Andreou et al., 2007; Kuntsi et al., 2005, 2006, 2009). For each condition (see below), a warning signal (four empty circles, arranged side by side) first appears on the screen. At the end of the fore‐period (presentation interval for the warning signal), the circle designated as the target signal for that trial is filled (coloured) in. The child is asked to make a compatible choice by pressing the response key that directly corresponds in position to the location of the target stimulus. Following a response, the stimuli disappear from the screen and a fixed intertrial interval of 2.5 s follows. Speed and accuracy are emphasised equally. The trial terminated if the child did not respond within 10 s.

The Fast task was completed in three sections. First, a practice session was administered, during which the child had to respond correctly to five consecutive trials. Second, the slow‐baseline condition was administered, consisting of 72 trials with an 8‐s fore‐period. Third and immediately following the slow‐baseline condition, the fast‐incentive condition was administered, consisting of 80 trials with a 1‐s fore‐period. For the fast‐incentive condition, the children were told to respond really quickly one after another, to win smiley faces and earn real prizes at the end. The children won a smiley face for responding faster than their own mean RT during the slow‐baseline (first) condition consecutively for three trials. The smiley faces appeared below the circles in the middle of the screen and were updated continuously. The fast‐incentive condition was always administered after the baseline condition and, as such, does not involve a similar learning phase. The children earned small prizes (vouchers) after completion of the fast‐incentive condition.

### Task performance parameters

Performance measures in the Fast task included errors of omission (failure to respond to the target) and commission (incorrect responses), reaction time to target stimuli (MRT, mean latency of responding in ms after target onset), within‐subject variability in reaction times (SD‐RT) and the coefficient of variation (CV, SD‐RT/MRT), calculated across correct responses that were detected between 200 and 1,500 ms poststimulus, excluding anticipations or excessively slow responses. RT data were also characterised using a mixture of the Gaussian and exponential distributions, called the ex‐Gaussian distribution (Luce, 1986). Mu and Sigma, measures of centrality and variability from the centre of the Gaussian curve, and Tau, a measure of centrality of the exponential component, were estimated from each individual RT data set using an iterative search‐based maximum likelihood estimation procedure (Lacouture & Cousineau, 2008). For analyses that compared performance across slow‐baseline and fast‐incentive conditions, data from 30 trials of the slow‐baseline condition were used, to provide a match on length of time on task with the fast‐incentive condition (findings were retained if all trials were used).

### Statistical analysis

One participant (one ASD + ADHD) was excluded from analysis due to extreme omission errors (<30% nonresponse to target in baseline condition), indicating lack of attention to task. In the slow‐baseline condition, MRT, Mu and Sigma were nonnormally distributed; in the fast‐incentive condition, MRT, SD‐RT, CV, Mu, Sigma and Tau were nonnormally distributed; and for difference scores calculated between conditions, MRT and CV were nonnormally distributed (indicated by sktest in Stata). Each of these parameters was therefore log‐transformed. The pattern of findings was retained for transformed and nontransformed data.

Groups were compared on performance in the slow‐baseline condition using multivariate ANOVA with group as the between‐subjects factor. Multiple comparisons were corrected using Sidak‐adjusted *p*‐values. A series of repeated‐measures ANOVAs were used to examine change in task performance from the slow‐baseline to the fast‐incentive condition. The task parameters investigated in the repeated‐measures analyses are limited to those that showed group differences in the slow‐baseline condition.

Age was a significant covariate for all parameters and was therefore retained in the analyses. The pattern of results was retained when IQ was entered as a covariate (see Supporting Information). The group analyses were performed in two stages: a comparison of four groups of TD, ASD, ADHD and ASD + ADHD to assess differences between pure and comorbid clinical groups; and 2 × 2 comparisons with ASD diagnostic status (ASD/ASD + ADHD vs. TDC/ADHD) and ADHD diagnostic status (ADHD/ASD + ADHD vs. TDC/ASD) entered as the between‐subjects factors. A nonsignificant interaction between the disorders is compatible with an additive model of comorbidity. Main effects and interactions at a significance level of *p* < .05 (two‐tailed) and trends (*p* < .10) were followed up with post hoc tests. Sidak correction was used to correct for multiple testing. Effect sizes (Cohen's *d*) were calculated using the difference in the means, divided by the pooled standard deviation of the data.

Where significant group differences were found, Spearman's correlations were conducted between the performance parameter and symptom scores. In order to account for the significant association between task performance parameters and age, as well as between ASD and ADHD symptoms in the sample (SCQ‐inattention: ρ = .38, *p* < .001; SCQ‐hyperactivity/impulsivity: ρ = .40, *p* < .001), these correlations were conducted on age and rating scale corrected scores. To correct for multiple correlations between rating scales and task performance parameters, Sidak‐adjusted *p*‐values were also applied. Differences in correlations between different performance measures were examined using Fisher's transformations.

## Results

### Group differences in task performance in slow‐baseline condition

Table 2 shows task performance in each group for slow‐baseline and fast‐incentive conditions.

**Table 2:** Nontransformed mean (*SD*) for task performance parameters in each group in the baseline and fast‐incentive conditions

	TDC	ASD	ADHD	ASD + ADHD
Baseline MRT	775.83 (201.44)	734.84 (202.40)	957.40 (276.14)	949.60 (220.39)
Baseline SD‐RT	200.65 (65.86)	239.08 (104.23)	354.86 (117.08)	324.66 (103.04)
Baseline CV	0.26 (0.06)	0.32 (0.10)	0.37 (0.08)	0.34 (0.07)
Baseline Mu	607.82 (163.12)	522.30 (138.18)	641.97 (253.59)	630.27 (103.04)
Baseline Sigma	93.78 (49.18)	74.38 (44.68)	136.62 (106.24)	100.18 (47.32)
Baseline Tau	167.01 (76.90)	212.55 (95.13)	315.42 (125.80)	319.33 (120.44)
Fast‐incentive MRT	303.67 (163.75)	558.43 (167.57)	616.36 (142.88)	626.47 (182.55)
Fast‐incentive SD‐RT	129.05 (70.09)	155.70 (77.27)	183.14 (69.04)	193.39 (104.15)
Fast‐incentive CV	0.22 (0.06)	0.27 (0.08)	0.29 (0.07)	0.30 (0.10)
Fast‐incentive Mu	443.61 (101.42)	424.85 (121.96)	464.19 (116.34)	455.68 (113.79)
Fast‐incentive Sigma	61.35 (35.90)	57.92 (41.59)	81.52 (50.91)	67.61 (33.03)
Fast‐incentive Tau	110.26 (72.29)	133.59 (73.86)	152.17 (65.54)	170.80 (103.86)

MRT, mean reaction time; SD‐RT, reaction time standard deviation; CV, coefficient of variation.

A significant multivariate effect of group emerged for task performance during the slow‐baseline condition [*F*(15, 246) = 3.02, *p* < .001; Pillai's trace = .47]. Using Sidak‐corrected *p*‐values, univariate testing indicated a significant effect of group on MRT [*F*(3, 84) = 5.13, *p* = .001], RTSD [*F*(3, 84) = 11.59, *p* < .001], Tau [*F*(3, 84) = 11.60, *p* < .001] and CV [*F*(3, 84) = 7.39, *p* = .02].

Compared to TDC, children with ADHD and ASD + ADHD were slower [ADHD: *p* = .02, *d* = 0.95, 95% CI = 0.32–1.58; ASD + ADHD: *p* = .01, *d* = 0.75, 95% CI = 0.21–1.31] and more variable in responding (ADHD – SD‐RT: *p* < .001, *d* = 1.62, 95% CI = 0.93–2.31; CV: *p* < .001, *d* = 1.07, 95% CI = 0.43–1.71; Tau: *p* < .001, *d* = 1.41, 95% CI = 0.74–2.08; ASD + ADHD – SD‐RT: *p* < .001, *d* = 1.34, 95% CI = 0.75–1.93; CV: *p* = .002, *d* = 0.90, 95% CI = 0.34–1.46; Tau: *p* < .001, *d* = 1.49, 95% CI = 0.89–2.09). In addition, children with ASD made faster responses compared to ADHD (*p* = .03, *d* = 0.77, 95% CI = 0.10–1.44) and ASD + ADHD (*p* = .02, *d* = 0.59, 95% CI = 0.01–1.19) and were less variable in their responses compared to ADHD (SD‐RT: *p* = .04, *d* = 0.95, 95% CI = 0.27–1.63) and ASD + ADHD (Tau: *p* = .04, *d* = 0.86, 95% CI = 0.25–1.47). There were no significant differences between ASD‐only and TDC (*p* > .05).

When combined by diagnosis, there was a significant effect of group for ADHD diagnosis (ADHD/ASD + ADHD) [*F*(5, 80) = 5.26, *p* < .001; Pillai's trace = .26]. Using Sidak‐corrected *p*‐values, univariate testing indicated a significantly greater MRT [*F*(1, 84) =  13.99, *p* = .001, *d* = 0.83, 95% CI = 0.40–1.26], SD‐RT [*F*(1, 84) = 26.91, *p* < .001, *d* = 1.14, 95% CI = 0.69–1.58], Tau [*F*(1, 84) = 25.91, *p* < .001, *d* = 1.17, 95% CI = 0.73–1.62] and CV [*F*(1, 84) = 13.28, *p* = .002, *d* = 0.87, 95% CI = 0.44–130] in children with ADHD (ADHD/ASD + ADHD) compared to children without ADHD (TDC/ASD). There was no multivariate effect of ASD diagnosis [*F*(5, 80) = 1.38, *p* = .24, Pillai's trace = .08]. There was a trend towards a significant interaction between ASD and ADHD diagnoses [*F*(5, 80) = 2.26, *p* = .06, Pillai's trace = .12], yet no performance measures met significance at Sidak‐corrected *p*‐values.

### Improvement of task performance in the fast‐incentive condition

**Figure 1:**
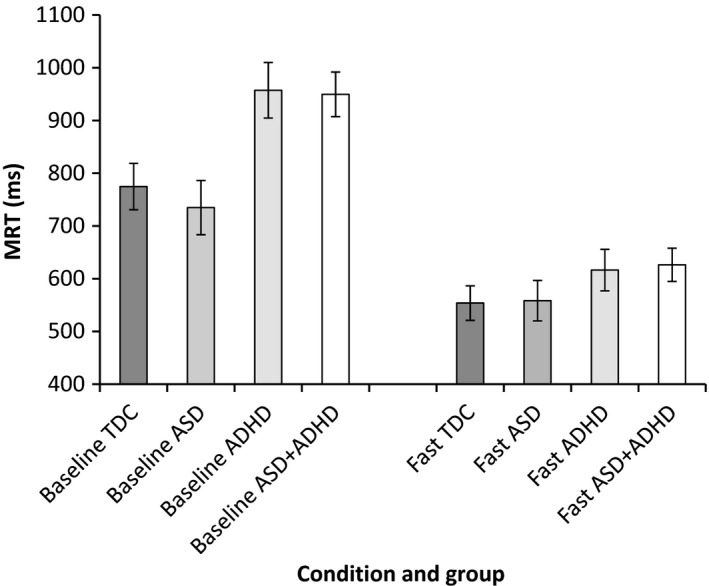
Mean reaction time (MRT) in baseline and fast‐incentive conditions for each group

**Figure 2:**
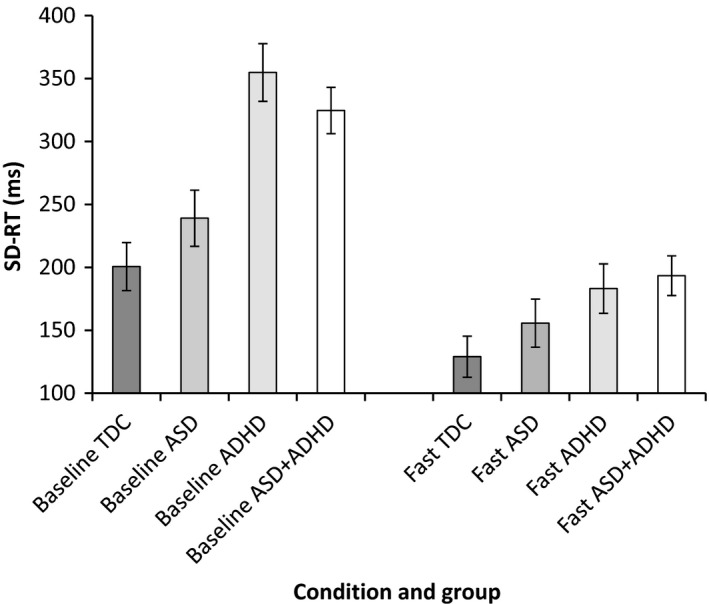
Standard deviation of reaction time (SD‐RT) in baseline and fast‐incentive conditions for each group

**Figure 3:**
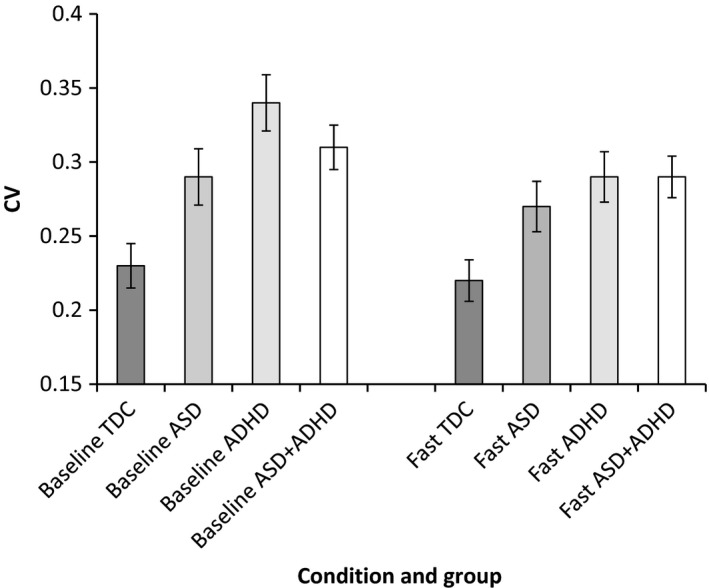
Coefficient of variation (CV) in baseline and fast‐incentive conditions for each group

**Figure 4:**
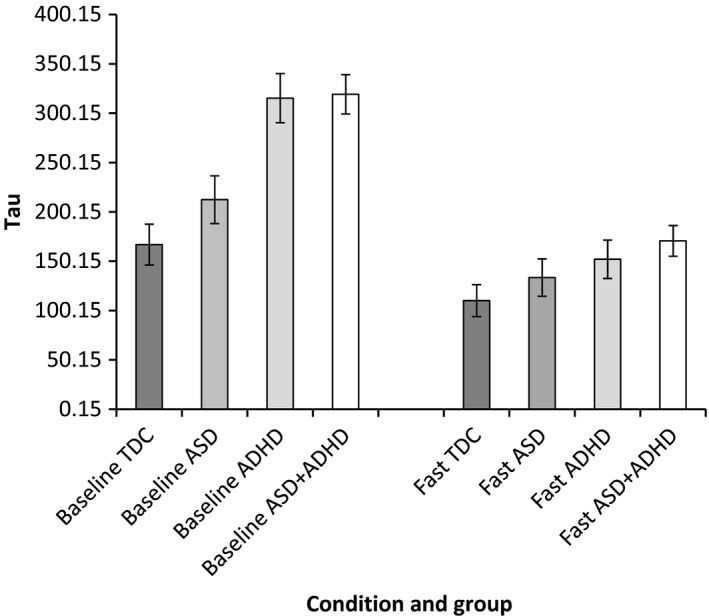
Tau in baseline and fast‐incentive conditions for each group

Task performance parameters showing significant group differences in the slow‐baseline condition were taken forward to examine change in task performance from slow‐baseline to fast‐incentive conditions using a series of repeated‐measure ANOVAs. See Figures 1, 2, 3, 4 for a graphical presentation of the findings.

#### MRT

There was a main effect of diagnostic group [*F*(3, 86) = 3.51, *p* = .02] and a significant interaction between group and condition [*F*(3, 86) = 6.52, *p* = .001]. Post hoc analyses revealed a significant difference by condition between (a) TDC and ADHD‐only (*p* = .04, *d* = 0.86, 95% CI = 0.26–1.51) (b) TDC and ASD + ADHD (*p* = .03, *d* = 079, 95% CI = 0.24–1.35), (c) ASD‐only and ADHD‐only (*p* = .007, *d* = 1.15, 95% CI = 0.46–1.85) and (d) ASD‐only and ASD + ADHD (*p* = .006, *d* = 1.06, 95% CI = 0.44–1.68), whereby there was a greater reduction in MRT from baseline to reward in the ADHD groups compared to the TDC and ASD‐only group (Figure 1). When combined by diagnostic status, there was a significant interaction between condition and ADHD diagnosis [*F*(1, 86) = 19.45, *p* < .001, *d* = 0.92, 95% CI = 0.49–1.35], indicating greater task performance improvement compared to children with no ADHD diagnosis. There was no interaction between condition and ASD diagnosis [*F*(1, 86) = 0.64, *p* = .43] and no interaction between ADHD and ASD diagnosis with condition [*F*(1, 86) = 0.17, *p* = .68].

#### SD‐RT

There was a main effect of diagnostic group [*F*(3, 86) = 8.51, *p* < .001] and a significant interaction between group and condition [*F*(3, 86) = 7.81, *p* < .001]. Post hoc analyses revealed a significant difference by condition between (a) TDC and ADHD‐only (*p* < .001, *d* = 1.27, 95% CI = 0.62–1.93); (b) TDC and ASD + ADHD (*p* = .03, *d* = 0.81, 95% CI = 0.26–1.37); and (c) ASD‐only and ADHD‐only (*p* = .002, *d* = 1.25, 95% CI = 0.54–1.95), indicating a greater reduction in RTSD from baseline to fast‐incentive conditions in the ADHD groups (see Figure 2). Trend‐level differences were also revealed between ASD‐only and ASD + ADHD (*p* = .08). When combined by diagnostic status, there was a significant interaction between condition and ADHD diagnosis [*F*(1, 86) = 22.15, *p* < .001, *d* = 0.97, 95% CI = 0.54–1.41], indicating greater task performance improvement compared to children with no ADHD diagnosis. There was no interaction between condition and ASD diagnosis [*F*(1, 86) = 1.07, *p* = .31] and no interaction between ADHD and ASD diagnosis with condition [*F*(1, 86) = 1.31, *p* = .26].

#### CV

There was a main effect of condition [*F*(1, 86) = 4.04, *p* = .05], indicating reduction in CV across all groups from baseline to fast‐incentive conditions. There was a main effect of diagnosis [*F*(3, 86) = 4.11, *p* = .05], but there was no interaction between group and condition on the CV [*F*(1, 86) = 0.99, *p* = .40; see Figure 3].

#### Tau

There was a main effect of diagnostic group [*F*(3, 86) = 6.02, *p* = .001] and a significant interaction between group and condition [*F*(3, 86) = 4.00, *p* = .01]. Post hoc analyses revealed a significant difference between TDC and ADHD‐only (*p* = .03, *d* = 0.89, 95% CI = 0.26–1.51), indicating a greater reduction in Tau from baseline to fast‐incentive conditions in the ADHD‐only group (see Figure 4). Trend‐level differences were also revealed between ASD‐only and ADHD‐only (*p* = .06, *d* = 0.87, 95% CI = 0.19–1.54). When combined by diagnostic status, there was a significant interaction between condition and ADHD diagnosis [*F*(1, 86) = 11.62, *p* = .001, *d* = 0.71, 95% CI = 0.28–1.13], indicating greater task performance improvement compared to children with no ADHD diagnosis. There was no interaction between condition and ASD diagnosis [*F*(1, 86) = 0.48, *p* = .49] and no interaction between ADHD and ASD diagnosis with condition [*F*(1, 85) = 0.38, *p* = .54].

### Dimensional analyses

Table 3 lists correlations between task performance parameters that indicated significant group differences and symptom scores for ASD and ADHD across the whole sample. In the baseline condition, there were significant positive correlations between ADHD symptoms and SD‐RT (inattention *r* = .35, *p* = .001, Sidak‐corrected *p* = .007; hyperactivity/impulsivity *r* = .30, *p* = .004, Sidak‐corrected *p* = .08), CV (inattention *r* = .37, *p* < .001, Sidak‐corrected *p* = .002; hyperactivity/impulsivity *r* = .36, *p* < .001, Sidak‐corrected *p* = .007) and Tau (inattention *r* = .30, *p* = .004, Sidak‐corrected *p* = .08; hyperactivity/impulsivity *r* = .28, *p* = .01, Sidak‐corrected *p* = .16), with a trend for MRT (inattention: *r* = .19, *p* = .08). Difference scores calculated between baseline and fast‐incentive conditions indicated significant correlations with ADHD symptoms and SD‐RT (inattention *r* = .39, *p* < .001, Sidak‐corrected *p* = .004; hyperactivity/impulsivity *r* = .29, *p* = .005, Sidak‐corrected *p* = .09) and Tau (inattention *r* = .25, *p* = .02, Sidak‐corrected *p* = .32; hyperactivity/impulsivity: *r* = .23, *p* = .03, Sidak‐corrected *p* = .48); with a trend for MRT (inattention *r* = .18, *p* = .09; hyperactivity/impulsivity *r* = .17, *p* = .09), Fisher's transformations indicated trend‐level differences in correlations between performance measures for (a) inattention and MRT versus inattention and CV (*z* = −1.37, *p* = .08); (b) inattention and MRT improvement versus inattention and SD‐RT improvement (*z* = −1.52, *p* = .06); and (c) hyperactivity/impulsivity and MRT versus hyperactivity/impulsivity and CV (*z* = −1.57, *p* = .06). There were no significant correlations between performance measures and ASD symptoms.

**Table 3:** Correlations between symptom scores and task performance parameters that indicated significant group differences, on age and rating scale corrected residuals

	Autism	Inattention	Hyperactivity/Impulsivity
Baseline MRT	.06	.19∧	.14
Baseline RTSD	.09	***.35*** **	.30**
Baseline CV	.07	***.37*** ***	***.36*** ***
Baseline Tau	.11	.30**	.28**
MRT improvement	−.03	.18∧	.17∧
RTSD improvement	−.07	***.39*** ***	.29**
Tau improvement	−.04	.25*	.23*

MRT, mean reaction time; RTSD, reaction time standard deviation; CV, coefficient of variation.

A positive correlation for improvement parameters indicates reduction from baseline to fast‐incentive conditions for the parameter of interest.

Key: *p* = ^∧^.09; *.05; ***.01; ***.001.

Bold italics = survive Sidak‐correction for multiple testing.

## Discussion

This study investigated task performance across slow and fast‐incentive conditions in children with ASD, ADHD and co‐occurring ASD + ADHD, compared to typically developing controls, using a task that has been used in several previous studies of ADHD. Findings from categorical diagnoses and quantitative trait measures converge to suggest longer and more variable RTs are specific to ADHD in slower conditions compared to ASD, while no significant group differences are observed in faster and more rewarding conditions. Children with ADHD also demonstrated greater reduction in response speed and variability from baseline to fast‐incentive conditions in comparison to children with ASD and typically developing controls. Those with comorbid ASD + ADHD were largely similar in performance to children with ADHD, and analyses support an additive co‐occurrence of the unique disorders.

The observation of longer and more variable RTs in children with ADHD (ADHD and ASD + ADHD) in the slow‐baseline condition, compared with children with ASD‐only and controls, is consistent with several previous studies that indicate the standard deviation of RT is a key marker of risk for the disorder (for review, see Karalunas et al., 2014; Klein, Wendling, Huettner, Ruder, & Peper, 2006; Kofler et al., 2013) and captures a proportion of the genetic risk for ADHD (Kuntsi et al., 2010). Significant associations with ADHD symptoms, particularly inattention when corrected for multiple comparisons, are consistent with recent research supporting greater genetic overlap between RTV with inattention compared to hyperactivity/impulsivity (Kuntsi et al., 2014), although significant associations between CV and hyperactive/impulsive symptoms were also demonstrated. The association with ADHD symptoms was stronger for RTV (SD‐RT and CV) than mean RT at a trend level, although firm conclusions regarding relative strength of association cannot be made. In addition, studies indicate response slowing and response variability form a distinct genetic factor (Kuntsi et al., 2010) and therefore likely reflect similar underlying processes (Klein et al., 2006). We extended these analyses to ex‐Gaussian parameters and demonstrated that increased Tau is associated with ADHD, which indicates that RT findings may reflect infrequent abnormally long RTs (Leth‐Steensen et al., 2000). Increased Tau did not, however, discriminate ADHD and control groups better than SD‐RT, in support of a recent meta‐analysis (Kofler et al., 2013). Importantly, RTV significantly discriminated ADHD‐only and ASD‐only in the slow‐baseline condition, indicating RTV in this nonarousing condition that is specific to the ADHD group.

A key finding is the sensitivity of performance in the ADHD groups to faster event rates and rewards. Children with ADHD (ADHD/ASD + ADHD) showed greater improvement in speed and variability of RT from the baseline to fast‐incentive condition, consistent with several studies (Andreou et al., 2007; Kuntsi et al., 2009; Slusarek et al., 2001; Van der Meere, Stemerdink, & Gunning, 1995). These findings support theoretical models that predict fluctuating cognitive performance dependent on energetic factors and reward in ADHD. It is important to note that slow event rates and lack of rewards do not necessarily account for increased RTV in ADHD. A recent meta‐analysis indicates effects of event rate on RT but not RT variability (Metin, Roeyers, Wiersema, van der Meere, & Sonuga‐Barke, 2012), yet this analysis did not explore the effect of rewards combined with event rate as optimising state factors. The performance of the ADHD groups did not completely reach the level of the control group, as indicated by the retention of significant group differences in the repeated‐measure analyses, which may suggest that optimal arousal had not been reached, for example due to the lengthy testing session, or alternatively that additional processes are involved (Willcutt, Doyle, Nigg, Faraone, & Pennington, 2005).

Importantly, the results indicate that event rate and rewards may not influence the performance of children with ASD, which suggests a different underlying neural process or correlate of RTV. One potential correlated process is activity related to the default‐mode network (DMN), which exhibits greater activity during rest and shows strong negative correlations with ‘task‐positive’ networks that are involved in task performance. For example, in children with ADHD, deactivation of the DMN from rest‐to‐task is increased when offered high incentives compared to low incentives (Liddle et al., 2011). Future work should directly compare neural processes underlying RTV in this design.

Taken together, the findings suggest that different properties of RTV and the conditions under which they are measured may aid in the characterisation of cognitive profiles in children with ASD and ADHD. These analyses suggest that increased RTV indexes liability for high ADHD symptoms across diagnostic boundaries, providing support for a transdiagnostic phenotype (Nolen‐Hoeksema & Watkins, 2011). The results also provide insight into the cognitive basis of the comorbidity between ASD and ADHD; the deficits exhibited in children with ASD + ADHD were generally compatible with an additive co‐occurrence showing the unique (and for several parameters more pronounced) deficits of both disorders, in line with findings from twin studies (Ronald, Simonoff, Kuntsi, Asherson, & Plomin, 2008) and examination of event‐related potentials (Tye, Asherson, et al., 2014; Tye, Battaglia, et al., 2014; Tye et al., 2013). It is important to note, however, that that nonadditive effects may be observed in a larger sample with increased power, as effects on the CV in the slow‐baseline condition did not survive multiple testing correction. Our previous work has suggested a nonadditive effect of ASD and ADHD diagnosis on CV during a continuous performance test (CPT; Tye, Asherson, et al., 2014), which implies distinct processes underlie cognitive performance in the comorbid group, as this group may show unique features that are not attributable to the joint influence of ASD and ADHD. This highlights the importance of comprehensive measurement of comorbid symptoms and warrants examination of the neural factors underlying RTV within genetically sensitive designs in order to elucidate the causal pathway. In particular, using the temporal precision of electroencephalography (EEG) will enable an investigation of the neurophysiological state preceding or correlated with variable behavioural responses (e.g. McLoughlin, Palmer, Rijsdijk, & Makeig, 2014).

Certain limitations should be taken into consideration. The relatively small sample size poses difficulties in the interpretation of the data and limits firm conclusions being made. The effect of intellectual ability should be considered: children with ADHD had lower IQ compared to typically developing children, and although not significantly different, IQ in the ASD group is relatively high, which may limit the generalisability of the findings. The pattern of results was largely retained when IQ was included as a covariate (see Supporting Information), yet four‐group comparisons of Tau improvement were not significant. Future work should directly examine the impact of intellectual ability according to the different conditions, particularly as discrepancies in IQ and other skills differ between these diagnostic groups (Ashwood et al., 2015). Still, a recent meta‐analysis of RTV in ASD‐control comparisons suggested that IQ is not a significant moderator of the association (Karalunas et al., 2014). Similarly, aetiological influences on ADHD and IQ are largely separate from other cognitive impairments in ADHD (Wood, Asherson, Van der Meere, & Kuntsi, 2010). Nevertheless, for individuals without intellectual impairments, cognitive deficits may be overlooked partly due to the employment of compensatory strategies or existence of protective mechanisms (Johnson, 2012). Future studies in substantial sample sizes are required to clarify the overlap in task performance in ASD and ADHD. The design of the task limits any differentiation between the effect of incentives and the effect of event rate, which may differentially affect the task performance measures examined (Epstein et al., 2011), and be mediated by separable brain processes (Rubia, 2011). Still, it has been demonstrated that RTV measured during slow‐baseline conditions, fast conditions and rewarded conditions, are indices of largely the same underlying aetiological process (Kuntsi et al., 2013). In addition, the effect of different types of reward remains unclear; for example, both groups demonstrate larger effects of monetary reward compared to social reward (Demurie et al., 2011). The smiley faces presented as indicators of reward in the current study may therefore affect performance.

## Conclusion

This is the first study to identify disorder‐specific and disorder‐convergent measures of RTV in children with ASD, ADHD and ASD + ADHD under varying conditions and differing indices of cognitive performance. The overlapping cognitive deficits may help to delineate shared aetiological underpinnings of ASD and ADHD, and the specific deficits may indicate why some children develop ADHD alone. Efforts to further refine the common and distinct processes underlying these complex disorders will likely aid in the identification of causal pathways as well as the design of more specific treatment strategies.

**Key points**

- Increased response time variability (RTV) may be a transdiagnostic phenotype of ASD and ADHD.
- We compared task performance in children with ASD, ADHD and co‐occurring ASD + ADHD during slow and fast‐incentive task conditions, using standard and ex‐Gaussian analyses.
- Longer and more variable RTs were demonstrated in ADHD and ASD + ADHD in both conditions.
- Children with ADHD and ASD + ADHD demonstrated greater improvement in performance from slow to fast‐incentive conditions.
- Increased RTV and improvement under optimal conditions may be specific to ADHD.

## Supporting information


**Appendix S1.** Analyses with age and IQ included as covariates.
**Table S1.** Correlations between RT parameters in baseline condition.
**Table S2.** Correlations between RT parameters in fast‐incentive condition.
