# Supplementary material for: Response time variability under slow and fast‐incentive conditions in children with ASD, ADHD and ASD+ADHD
Source: J Child Psychol Psychiatry. 2016 Jul 28;57(12):1414–23. doi: 10.1111/jcpp.12608 (PMC5132150; doi:10.1111/jcpp.12608)
Supplement: Supplementary file 1 — Appendix S1. Analyses with age and IQ included as covariates. Table S1. Correlations between RT parameters in baseline condition. Table S2. Correlations between RT parameters in fast‐incentive condition. [file JCPP-57-1414-s001.docx]

**Supplementary Material**

**Response time variability under slow and fast-incentive conditions in children with ASD, ADHD and ASD+ADHD**

**Appendix S1.** Analyses with age and IQ included as covariates.

A significant multivariate effect on group emerged for task performance during the slow-baseline condition [F (15, 243) =2.49, p=.002; Pillai’s trace=.40]. Using Sidak-corrected p-values, univariate testing indicated a significant effect of group on MRT [F(3,83) = 3.29, p=.04], RTSD [F(3,83) = 7.78, p<.001], Tau [F(3,83) = 8.47, p<.001] and CV [F(3,83) = 5.04, p=.01].

Compared to TDC, children with ADHD were slower [ASD+ADHD: p=.04, d=0.78, 95% CI=-0.23-1.33] and more variable in responding (ADHD - SD-RT: p<.001, d=1.38; CV: p=.006, d=1.11; Tau: p=.003, d=1.20; ASD+ADHD – SD-RT: p<.001, d=1.20; CV: p=.01, d=0.96; Tau: p<.001, d=1.37). In addition, there was a trend for children with ASD to be less variable in their responses compared to ASD+ADHD (Tau: p=.09, d=0.77). There were no significant differences between ASD-only and TDC (all p>.05).

When combined by diagnosis, there was a significant effect of group for ADHD diagnosis (ADHD/ASD+ADHD) [F (5, 79) =3.76, p=.004; Pillai’s trace=.19]. Using Sidak-corrected p-values, univariate testing indicated a significantly greater MRT [F(1,83) = 8.66, p=.04, d=0.69], RTSD [F(1,83) = 16.81, p<.001, d=0.96], Tau [F(1,83) = 17.17, p<.001, d=1.01] and CV [F(1,83) = 7.49, p=.03, d=0.75] in children with ADHD (ADHD/ASD+ADHD) compared to children without ADHD (TDC/ASD). There was no multivariate effect of ASD diagnosis [F (5,79) = 1.42, p=.23, Pillai’s trace=.08]. There was a trend towards a significant interaction between ASD and ADHD diagnoses [F (5,79) = 2.06, p=.08, Pillai’s trace=.12), yet no performance measures met significance at Sidak-corrected p-values.

#### Improvement in the fast-incentive condition

*MRT*

There was a significant interaction between group and condition [F (3,85) = 6.31, p=.001). Post-hoc analyses revealed a significant difference between (i) TDC and ADHD-only (p=.03, d=0.95) (ii) TDC and ASD+ADHD (p=.02, d=0.84), (iii) ASD-only and ADHD-only (p=.006, d=1.19) and (iv) ASD-only and ASD+ADHD (p=.005, d=1.09), whereby there was a greater reduction in MRT from baseline to reward in the ADHD groups compared to the TDC and ASD-only group. When combined by diagnostic status, there was a significant interaction between condition and ADHD diagnosis [F (1,85) = 18.60, p<.001, d=0.95), indicating greater task performance improvement compared to children with no ADHD diagnosis. There was no interaction between condition and ASD diagnosis [F (1,85) = 0.75, p=.39] and no interaction between ADHD and ASD diagnosis with condition [F (1,85) = 0.09, p=.77].

*SD-RT*

There was a main effect of diagnostic group [F (3,85) = 5.48, p=.002) and a significant interaction between group and condition [F (3,85) = 5.11, p=.003). Post-hoc analyses revealed a significantly difference between TDC and ADHD-only (p=.01, d=1.12) and ASD-only and ADHD-only (p=.01, d=1.10), indicating a greater reduction in RTSD from baseline to fast-incentive conditions in the ADHD-only group. Trend-level differences were also revealed between TDC and ASD+ADHD (p=.08). When combined by diagnostic status, there was a significant interaction between condition and ADHD diagnosis [F (1,85) = 14.73, p<.001, d=0.80), indicating greater task performance improvement compared to children with no ADHD diagnosis. There was no interaction between condition and ASD diagnosis [F (1,85) = 0.89, p=.35] and no interaction between ADHD and ASD diagnosis with condition [F (1,85) = 1.00, p=.32].

*CV*

There was no significant main effects or interactions on the CV (all p>.05).

*Tau*

There was a main effect of diagnostic group [F (3,85) = 4.12, p=.009) and a trend towards an interaction between group and condition [F (3,85) = 2.54, p=.06). Post-hoc analyses revealed no significant group differences by condition. When combined by diagnostic status, there was a significant interaction between condition and ADHD diagnosis [F (1,85) = 7.49 p=.01, d=0.60), indicating greater task performance improvement compared to children with no ADHD diagnosis. There was no interaction between condition and ASD diagnosis [F (1,85)=0.38, p=.54] and no interaction between ADHD and ASD diagnosis with condition [F (1,85) = 0.25, p=.62].

|  | MRT | SD-RT | CV | Mu | Sigma |
| --- | --- | --- | --- | --- | --- |
| SD-RT | .85*** |  |  |  |  |
| CV | .33** | .77*** |  |  |  |
| Mu | .85*** | .46*** | -.13 |  |  |
| Sigma | .73*** | .54*** | .13 | .74*** |  |
| Tau | .73*** | .92*** | .77*** | .33** | .36*** |

**Table S1**: Correlations between RT parameters in baseline condition

** p<.01 ***p<.001

|  | MRT | SD-RT | CV | Mu | Sigma |
| --- | --- | --- | --- | --- | --- |
| SD-RT | .87*** |  |  |  |  |
| CV | .59*** | .90*** |  |  |  |
| Mu | .89*** | .61*** | .25* |  |  |
| Sigma | .76*** | .65*** | .41*** | .82*** |  |
| Tau | .84*** | .95*** | .86*** | .53*** | .50*** |

**Table S2**: Correlations between RT parameters in fast-incentive condition

* p<.05 ** p<.01 ***p<.001
